# Editorial: Trauma, Psychosis and Posttraumatic Stress Disorder

**DOI:** 10.3389/fpsyt.2017.00220

**Published:** 2017-11-03

**Authors:** Kate V. Hardy, Kim T. Mueser

**Affiliations:** ^1^Department of Psychiatry and Behavioral Sciences, Stanford University, Stanford, CA, United States; ^2^Center for Psychiatric Rehabilitation, Boston University, Boston, MA, United States

**Keywords:** psychosis, posttraumatic stress disorder, trauma, treatment, childhood trauma

**Editorial on the Research Topic**

**Trauma, Psychosis and Posttraumatic Stress Disorder**

Exposure to psychologically traumatic experiences has been part of the human condition throughout history, but only within the last half century has research provided insight into the short- and long-term sequelae of trauma, ultimately resulting in the creation of a specific diagnostic category to capture the most common negative consequences. In 1980, posttraumatic stress disorder (PTSD) was included in the Diagnostic and Statistical Manual of Mental Disorders (DSM-III), setting the stage for research and clinical practice to more systematically study and treat this mental health problem. Since the inclusion of PTSD in DSM-III, there has been huge growth in the field’s understanding of PTSD, and the development of concomitant evidence-based treatments to aid individuals with PTSD in overcoming this disorder and returning to previous levels of functioning.

However, our understanding of the link between trauma and psychosis may still be considered nascent by comparison, despite burgeoning evidence for a clear link between childhood adverse experiences and psychotic symptoms (1–3). In addition, it is only relatively recently that clinical interventions designed to target trauma and its consequences in individuals presenting with psychosis have been developed. This may be in part due to an historic focus on biological explanations for the onset of psychotic disorders, but also clinicians’ perceived fear of “opening Pandora’s Box” by talking with individuals with psychotic symptoms about their traumatic experiences, and fearing a risk to stabilization and exacerbation of their symptoms (4). Lifetime prevalence rates of PTSD in individuals diagnosed with a psychotic disorder have been estimated at 30% compared with 7.8% in the general population (5), although this may be an underestimate as there is concern that trauma and PTSD goes unreported, and unrecognized, in individuals with serious mental illness (6). The link between psychosis and trauma is complex and multifactorial with different proposed pathways. These include (1) psychosis as a result of childhood adversity (3), (2) trauma as a result of psychotic symptoms or involuntary treatment experiences (7, 8), (3) psychosis as a dimension of PTSD resulting from trauma (9, 10), and (4) PTSD and retraumatization as stressors that can worsen the course of a psychotic disorder (11). In addition to the impact of trauma on the development of psychosis and PTSD, there is evidence that traumatic experiences influence the content of psychotic symptoms, including hallucinations and delusions (2, 12). However, the mechanisms involved in these pathways from trauma to psychosis and PTSD are not fully understood and different models have been posited to explicate this link.

The aim of this Research Topic is to collate a series of articles addressing PTSD, trauma, and psychosis across a variety of formats, including opinion pieces, reviews, and original research. In doing so, this Research Topic will assemble the latest data and expert opinion on the current state of research and clinical intervention in this rapidly growing field, as well as highlight potential future research directions. Three key sections will be explored in this Research Topic: (1) PTSD, including neurobiological understanding of risk for PTSD and proximal and longitudinal consequences of trauma exposure; (2) Trauma and Psychosis, including proposed mechanisms and pathways; and (3) Clinical Interventions, including empirical studies providing data on treatments. The overarching purpose of this Research Topic is to provide a better understanding of the interface between trauma, psychosis, and PTSD.

## Posttraumatic Stress Disorder

Furthering our understanding of PTSD is essential to exploring the link between trauma and psychosis. While the experience of traumatic events is unfortunately common, the majority of individuals who are exposed to trauma do not go on to develop PTSD. Of interest are the questions of why some people develop PTSD and whether it is possible to identify these individuals at an early stage to aid earlier targeted intervention. Increasing our understanding of the neurobiological mechanisms implicated in the development of PTSD can aid in this process. In this Research Topic, Wang et al. examine electrophysiological data to establish the presence of a physiological prodrome of PTSD in an attempt to identify possible markers that could identify opportunities for early treatment. Lee et al. address the question of the role of dopamine in the development of PTSD and propose the Rebound-Excitation Theory to explain the variability in stress resilience. Considering that dopamine has been hypothesized to play a central role in the pathophysiology of schizophrenia for over 50 years (13–15), further insight into the role of dopamine in PTSD may aid our understanding of the link between trauma and psychosis.

Conceptualizing PTSD across the time course is necessary to understand the development and maintenance of mental health problems over time. In this Research Topic, Ibrahim and Hassan examine data from Syrian Kurdish refugees living in a refugee camp and exposed to torture and other traumatic events, while Bovin et al. report on the longitudinal associations between PTSD severity and personality disorder features. Both these studies have important implications for when treatment is provided and its likely impact. Ibrahim and Hassan highlight the need for psychological services for Syrian Kurdish refugees that would potentially occur shortly after the traumatic event. Bovin et al., on the other hand, draw on their longitudinal data to demonstrate that improvements in PTSD symptoms are associated with improvements in characterological features (and vice versa), suggesting that targeting PTSD symptoms in individuals with a personality disorder may result in improvements in both psychopathology and comorbid personality traits over time. This time course (from shortly after the trauma to years later) indicates the need to conceptualize PTSD as longitudinal, and offer ongoing assessment and targeted treatments at different stages.

## Trauma and Psychosis

Of critical importance in this Research Topic is the inclusion of a first-person account of trauma and psychosis written from the perspective of someone with “lived experience” (Britz). The voices of persons with lived experience has been essential in our understanding of psychosis at multiple levels, but has been less widely explored in relation to trauma and psychosis (16). Britz writes eloquently, and with disarming honesty, about her experience of trauma and psychosis, and developing an understanding of the interface between the two while also drawing upon current discourse to highlight the importance of meaningful collaboration with people with lived experience. Adding to this lived experience perspective is Lu et al.’s qualitative analysis of posttraumatic reactions to psychosis that provides a narrative description of the key themes of the traumatizing nature of psychosis, including symptoms, treatment, and the corresponding emotional reactions to these.

As previously mentioned, although a clear link between trauma and psychosis has been established, the specific mechanisms involved are still unknown. Two papers in this research topic examine potential explanatory models. Berry et al. focus on a model specific to understanding the development of auditory hallucinations. This paper is the first to propose a theoretical link between early childhood attachment and dissociative processing as mechanisms to explain the origin, and maintenance of distressing voice hearing. Hardy proposes a comprehensive, theoretically informed model of posttraumatic stress in psychosis that encompasses emotion regulation and autobiographical memory to understand the pathway between victimization and psychosis and provides case vignettes to illustrate how this model informs case formulation and treatment. Brand et al. highlight the ethical challenges associated with experimental manipulation of possible causal pathways to scientifically establish links between trauma exposure, PTSD, and psychosis and propose an interventionist-causal paradigm to better understand this relationship. This approach examines the impact of an intervention on a proposed causal mechanism compared with a control intervention while observing the impact on the symptom of interest. The authors propose several potential mechanisms, including memory processing, negative posttraumatic beliefs, dissociation, and posttraumatic avoidance with connected interventions. This interventionist-causal paradigm has already been applied in psychosis research (17) and offers a model to better understand proposed mechanisms in trauma and psychosis.

Recognizing the debate regarding schizophrenia as a unitary diagnostic category, Stevens et al. propose four subgroups of trauma in psychosis in order to elaborate on symptom-specific conceptualizations of distress and propose corresponding interventions for these four subtypes. The concept of psychosis on a continuum, rather than as a discrete entity, is also of importance to the paper presented by Mayo et al., who address the role of psychosis and stressful life events in individuals determined to be at-risk of developing psychosis. This population is important to this topic in that they are a group of individuals who have not yet developed full psychosis, and may indeed not do so, but who are typically experiencing attenuated psychotic symptoms and are distressed and help seeking. Alarmingly, this population reports high levels of childhood trauma and the paper reviews these data while providing clinical recommendations on the assessment, treatment, and future research directions.

As previously discussed, there are concerns in the field about the under-detection of trauma in persons with psychosis or other severe mental illnesses. Under-detection due to professionals failing to screen for trauma and PTSD can be overcome by routine screening of individuals receiving services (18). Church et al. examine another potential factor contributing to poor identification of trauma history and its consequences in this population—the minimization or denial of childhood trauma by individuals themselves. In line with this theme of the importance of accurate assessment, Rosen et al. explored cumulative exposure of traumatic life events. In particular their use of qualitative analysis in the study highlights the importance of careful and sensitive assessment to understanding the time course and impact of trauma on individuals and their mental health.

The recovery literature has changed how recovery from mental illness is understood, with a shift from traditional medical definitions that emphasize symptom remission to conceptualizing recovery as a personally meaningful process that involves the development of meaning and a sense of purpose despite symptoms or other challenges (19, 20). In this Research Topic, Mazor et al. examine the experience of posttraumatic growth as mediated by meaning making and coping self-efficacy adding a much needed focus on resiliency in this population.

## Clinical Interventions

Interventions specifically for PTSD in individuals with a psychotic disorder are not as well established as for psychosis (e.g., cognitive behavioral therapy for Psychosis) or PTSD (e.g., cognitive processing therapy, eye movement desensitization and reprocessing therapy, prolonged exposure), although some recent progress has been made (21–23). Further work continues in this area with growing awareness of the link between trauma and psychosis and the need to provide targeted interventions that address the PTSD. In this Research Topic, Swan et al. provide a systematic review of interventions supporting the evidence that trauma-focused psychological interventions can be applied safely and effectively in individuals with psychosis. A range of interventions have been studied for trauma and psychosis and in this Research Topic Prolonged Exposure (Grubaugh et al.), Trauma Focused CBT (Keen et al.), and interventions specific to trauma and voices (Steel) are all discussed. However, despite emerging evidence that these interventions are safe and effective, there remains a challenge of dissemination. Cragin et al. begin to address this through the development of clinical practice guidelines to aid clinicians working with early psychosis and comorbid trauma-related disorders.

## Conclusion

The articles in this Research Topic demonstrate the breadth of current research being conducted in this field. The authors of the included articles further the discussion around the interface between trauma, psychosis, and PTSD and provide cogent arguments for future research and clinical application of the data presented. They collectively highlight the need to identify, assess, and address trauma in this population which for too long has been overlooked and under treated.

## Author Contributions

KH and KM co-edited this Research Topic and co-wrote the editorial.

## Conflict of Interest Statement

The authors declare that the research was conducted in the absence of any commercial or financial relationships that could be construed as a potential conflict of interest.
